# Fe(II)-activated persulfate oxidation to degrade iopamidol in water: parameters optimization and degradation paths

**DOI:** 10.1038/s41598-020-78468-y

**Published:** 2020-12-09

**Authors:** Zijun Dong, Guanhan Chen, Mu Li, Feiyun Sun, Chengchun Jiang, Bandna Bharti

**Affiliations:** 1 School of Civil and Environmental Engineering, Shenzhen Polytechnic, Shenzhen, 518055 China; 2 School of Civil and Environmental Engineering, Harbin Institute of Technology Shenzhen, Shenzhen, 518055 China

**Keywords:** Chemical engineering, Environmental chemistry

## Abstract

Iodinated contrast media (ICM), which was widely used in medical imaging and was difficult to remove by conventional wastewater treatment methods, attained much attention due to its potential environmental impacts. Herein, iopamidol (IPM), one typical compound of ICM, was found to be rapidly degraded by ferrous activated persulfate oxidation (Fe(II)/PS) as compared with PS or Fe(II) alone. With a persulfate concentration of 1 mmol L^−1^, n(Fe(II))/n(PS) of 1:10, and a pH of 3.0, 78% IPM was degraded within 60 min, with a degradation rate of 0.1266 min^−1^. It was demonstrated that IPM degradation and deiodination were favored by a high temperature, while affected positively by acidic and neutral conditions. Radical quenching experiments and Electron Paramagnetic Resonace (EPR) spectra showed that the combined effects of SO_4_^−^**·** and **·**OH contributed dominantly to degrade IPM, while the **·**OH played an essential role during the degradation reaction. Through the Discrete Fourier Transform quantum chemical calculation, the possible reaction pathways for the oxidation of IPM by **·**OH are as follows: IPM-TP651-TP667-TP541-TP557, IPM-TP651-TP525-TP557, IPM-TP705-TP631-TP661, and IPM-TP705-TP735. The obtained results showed that IPM could be degraded effectively by Fe(II)/PS system, giving a promising technique for IPM removal from water.

## Introduction

Iodinated X-ray contrast media (ICM) has been widely used at medical centres and hospitals as an intravascular pharamaceutical to enhance the imaging of human organs, blood vessels and tissues^1^. Although ICM is non-toxic to human bodies, it is the source of total adsorbable organic iodine (AOI) in an aqueous environment that can act as a precursors to highly toxic iodinated disinfection by-products (I-DBPs)^2^. Creation of harmful I-DBPs is a progressing concern for the utilities of water. Generally, I-DBPs are considered to be more genotoxic and cytotoxic as comapared to their brominated and chlorinated additives^3,4^. ICM is frequently detected in aquatic systems, for instance, in China the total concentration of ICM was found to be between 102 to 252 ng L^−1^ in Huangpu River and 88.7 to 131 ng L^−1^ in Taihu Lake^5^. Specifically, iopamidol (IPM), a typical ICM compound, was found in water resources with a concentration of about 1900 ng L^−1^ in Japan^6^. The growing IPM concentration has been traced in surface water, ground water, hospitals, treated wastewater effluents and domestic wastewater. As the IPM is one of the major contributor to I-DBPs formation^7^, and chlorination of I^−^ could quickly form HOI/I_2_, which was react with natural organic matters to form I-DBPs^8^. The odour and taste problems in drinking water were caused by I-DBPs.

Therfore, removal of IPM form the aquatic system have attained much attention. Various types of physical, chemical and biochemical techniques have been reported for the removal of IPM. From an economic and efficiency viewpoint and the biological recalcitrance of IPM^9^, researchers mainly focused on adopting physicochemical methods, rather than using single-stage physical, chemical or biochemical methods to degrade IPM in water. For example, Kong et al. studied the degradation of IPM by using UV/chlorine, by comparing with UV or chlorine alone^10^, and they found that in contrast to chemical processes, biochemical methods have limited pollutants removal efficiency. For instance, it took over 20 days to achieve more than 95% of IPM degradation by using anaerobic process^11^.

Recent reports on IPM removal and degradation by several treatment techniques were summarized in Table 1. It was found that both Fe(VI) oxidation and UV/chlorine process had a relative high IPM degradation rate among these processes, although which were limited by their higher cost and potential production of DBPs. Interestingly, persulfate (PS) or peroxymonosulfate (PMS) based processes have the high rate constant and removal rate to deal with IPM among these processes as shown in Table 1. PS was used to generate sulfate radicals (SO_4_^−^**·**) by either homogeneous or heterogeneous advanced oxidation process (AOPs). Being relatively stable at a room temperature, PS can be activated by several means, such as heat^12^, transition metals^14,15^, to generate SO_4-_^−^·. However, some PS based technology, such as PS/ZVA system^13^, PS/UV-A process^16^, have its own drawbacks, such as precise pH condition, and not eco-friendly, e.g. in the CuO/PMS system^14^. On the other hand, sometimes additional complexion agents were required that would increase the activation efficiency, such as PS/Fe(III)/GA system, but sidewise they may also increase the operational cost. Therefore, it is necessary to develop an economical technology that could degrade IPM under a neutral and mild conditions.

**Table 1:** IPM main treatment process (2016–2018).

Process	IPM concentration (μM)	Treatment condition parameters	IPM degradation efficiency (%)	Rate Constant	Ref
CuO/PMS^a^ system	2.6	Temp = 25 ± 2 °C; pH = 7.0; [PMS] = 100.0 mg L^−1^; CuO dose = 0.2 g L^−1^	100 (in 15 min)	0.218 min^−1^	^14^
Fe(VI) oxidation	10	Temp = 25 °C; pH = 7.0 [Fe(VI)] = 0.5 mM	> 80 (in 60 min)	55.8 ± 3.5 M^−1^ s^−1^	^17^
Chlorine	5	Temp = 25 ± 1 ℃; pH = 7.0 [Cl_2_] = 200 μM	–	(1.66 ± 0.09) × 10^–3^ M^−1^ s^−1^	^18^
UV	2	Temp = 23 ± 1 °C; pH = 7.0	< 10 (in 30 min)	0.0336 min^−1^	^10^
UV/chlorine	2	Temp = 23 ± 1 °C; pH = 7.0; chlorine dosage = 200 μM Light source: 0.13 mW cm^−2^	–	0.3456 min^−1^	^10^
Photocatalytic treatment processes using TiO_2_	25.7	TiO_2_ Dosage = 1000 mg L^−1^ Light source: 40 W, λ < 360 nm	100 (in 16 h)	–	^19^
PS^b^/UV-A process	2.6	[PS] = 0.5 mM; pH = 11; Light source: UV-A, 7.6 W m^−2^	100 (in 60 min)	0.1535 ± 0.0037 min^−1^	^16^
PS/ZVA^c^ system	2.6	pH = 3; ZVA = 1 g/L; PS = 0.5 mM;	55 (in 60 min)	–	^13^
PS/Fe(III)/GA^d^ system	20	[PS] = 0.2 mM; [Fe(III)] = 10 μM; [GA] = 10 μM; pH = 7.0; T = 25 °C	70 (in 60 min)	0.048 min^−1^	^20^
ZnO-based materials and enzymes hybrid systems	12.9	Photocatalyst (SMA-Ce-ZnO-plus SBP) dosage = 1 g L^−1^; Light source: 40w, λ < 360 nm; solution: 5 mL, pH = 5.4	70 (in 24 h)	1.2 × 10^−3^ min^−1^	^15^
Anaerobic transformation	2.6	Oxygen-free Rhine water (25 mL) was added to 10 g of anaerobic sediment taken from a sulfate-reducing zone of a polishing pond	< 5 (in 60 min)	–	^11^
Solar Photocatalytic Degradation with Bi(0)-Doped Bismuth Oxyhalide Thin Films	0.13	Catalysis = 3%Bi-doped BiOCl_0.875_Br_0.125_ films Light source: 500 W m^−2^ 280–950 nm	55 (in 60 min)	–	^9^
ZVA activated persulfate	2.6	ZVA = 1 g L^−1^; PS = 0.50 Mm; pH = 3; T = 25 °C	52 (in 60 min)	–	^21^
Photocatalytic treatment with Ce-doped ZnO	25.7	[Ce-doped ZnO] = 1000 mg /L; λ = 290–400 nm (Intensity = 24 ± 1 W·m^−2^); Temp = 26 °C	100 (in 30 min)	0.12 min^−1^	^22^
Electrochemical treatment with BDD^e^ electrodes	19.3–32.2	Na_2_SO_4_ = 2 mS/cm; 0.31 mA/cm^2^; Temp = 20 °C	> 90 (in 16 h)	1.2 × 10^–3^ min^−1^	^23^

a. PMS = Peroxymonosulfate.

b. PS = Persulfate.

c. ZVA = Zero-valent Aluminum.

d. GA = Gallic acid.

e. BDD = Boron-doped diamond (BDD) electrodes.

Among these activators, transition metal Fe(II) shows high activity to initiate PS decomposition, as it could be injected directly in subsurface that promotes in-situ oxidation^24^. Especially, owning to its merits, e.g. non-toxic, environmental friendly and relatively low cost, Fe(II) is deemed as one of the most promising activators^25^. Zhu et al. established a kinetic model to describe the iohexol degradation in the Fe(II) activated PS system, and they found that the rate constant of iohexol reacting with sulfate radical was (1.83 ± 0.10) × 10^9^ ^26^. Bu et al. proposed a mathematical model on Fe(II)- activated PS oxidation of atrazine (ATZ), and they observed that only small amount of **·**OH were produced while SO_4_^−^**·** attributed a certain level to ATZ degradation in Fe(II)/PS system^27^.

However, to the best of our knowledge, the investigation on IPM degradation by Fe(II)/PS are rarely reported, up to now. In the present study, Fe(II)/PS oxidation was employed to degrade IPM, and the influence of operational condition parameters on the IPM treatment efficiency was examined to obtain the optimized IPM removal condition parameters. Meanwhile, the IPM degradation pathway was also examined computationally. The obtained results will be very useful for IPM degradation and removal in water and wastewater.

## Materials and methods

### Experimental setup and design

All chemicals used in this study were analytical grade. Iopamiol hydrate (IPM, 98%) was purchased from Meilun (China), and sodium persulfate (Na_2_S_2_O_8_, 99%) was purchased from Aladdin (China). Ferrous sulfate heptahydrate (FeSO_4_⋅7H_2_O, 99.0–101.0%) and sodium carbonate anhydrous (Na_2_CO_3_) was purchased from XILONG Chemical CO., LTD. China and Tianjin Mao Tai Chemical Reagent Factory, respectively. Tertiary butyl alcohol (TBA) was purchased from Damao Chemical Reagent Factory, and high performance liquid chromatography (HPLC) grade methanol (MeOH, ≥ 99.9%) was supplied by Merck KGaA.

The experiment was performed in a 250 mL reagent bottle with a working volume of 100 mL, which was installed in an electro-thermal shaking chamber (Shanghai Bluepard Instrument, Co. Ltd., China). During the experiment, 10 μM IPM was first prepared with distilled water (Shanghai Hitech Instruments Co., LTD, China). Pre-determined amount of ferrous sulfate solution was added to the reagent bottle. The initial pH was adjusted with 0.1 M sulfate acid and 0.1 M sodium hydroxide solution, and its temperature was kept at a constant value throughout the reaction. Afterwards, a certain amount of PS (0.1 M) was added. With the progression of reaction, 0.5 mL mixture in the bottle was accurately sampled at selected time intervals (0, 5, 10, 30 and 60 min). Afterwards, the reaction was quenched intermediately by using sufficient volume of methanol, which is an effective scavenger to quench SO_4_^−^· and ·OH to stop oxidation reactions. Besides, Iodide ((I^−^) and iodate (IO_3_^−^) were analyzed after treatment.

The effect of Fe(II) activated PS oxidation process parameters on IPM degradation was investigated by comparison with a blank experiment. Three parallel experiments were carried out, respectively, by adding 1 mM PS, 0.1 mM Fe(II), and Fe(II)/PS, after that all of them were added and reacted at a pH of 3 at 25 °C.

### Analytical procedures

The concentration of IPM in the solution was determined with an Alliance 2695 Series high-performance liquid chromatography (HPLC) system equipped with a C18 column (5 μm, 4.6 × 20 mm) at a UV wavelength of 242 nm. The column temperature was maintained at 35 °C, and the mobile phase was a mixture of methanol/water (60/40, v/v) with a flow rate of 1.0 mL min^−1^. The injection volume was kept at 100 μL. Iodide (I-) and iodate (IO_3_^−^) were determined by ion chromatography (IC, Thermo Dionex ICS-1500). The sample quenched by sodium sulfite were filtered by BOND ELUTE C18 to remove any organic matters, and then were injected into IC equipped with a IonPacAS9-HC column (250 mm × 4 mm). The mobile phase was 9 mM sodium carbonate and run at a flow of 1 mL min^−1^.

### Computational methods

The chemical calculations are performed in the framework of Discrete Fourier Transform (DFT) using Gaussian 09 package according to the previous report^28^. The computational accuracy, feasibility and economical computational time were considered when choosing the computational levels and basis sets. The geometrical parameters were optimized at B3LYP level with a standard of 6-31G* basis set. Regarding basis set, iodine was defined to fifth cycle element, making the calculations computationally intensive. The use of relativistic effective core potentials is necessary to make the calculations tractable while obtaining accurate theoretical single point energies. Specially, B3LYP /SDD was used for I atom, and the 6-31G* basis set was used for other elements.

## Results and discussion

### IPM degradation by Fe(II)/PS oxidation

**Figure 1:**
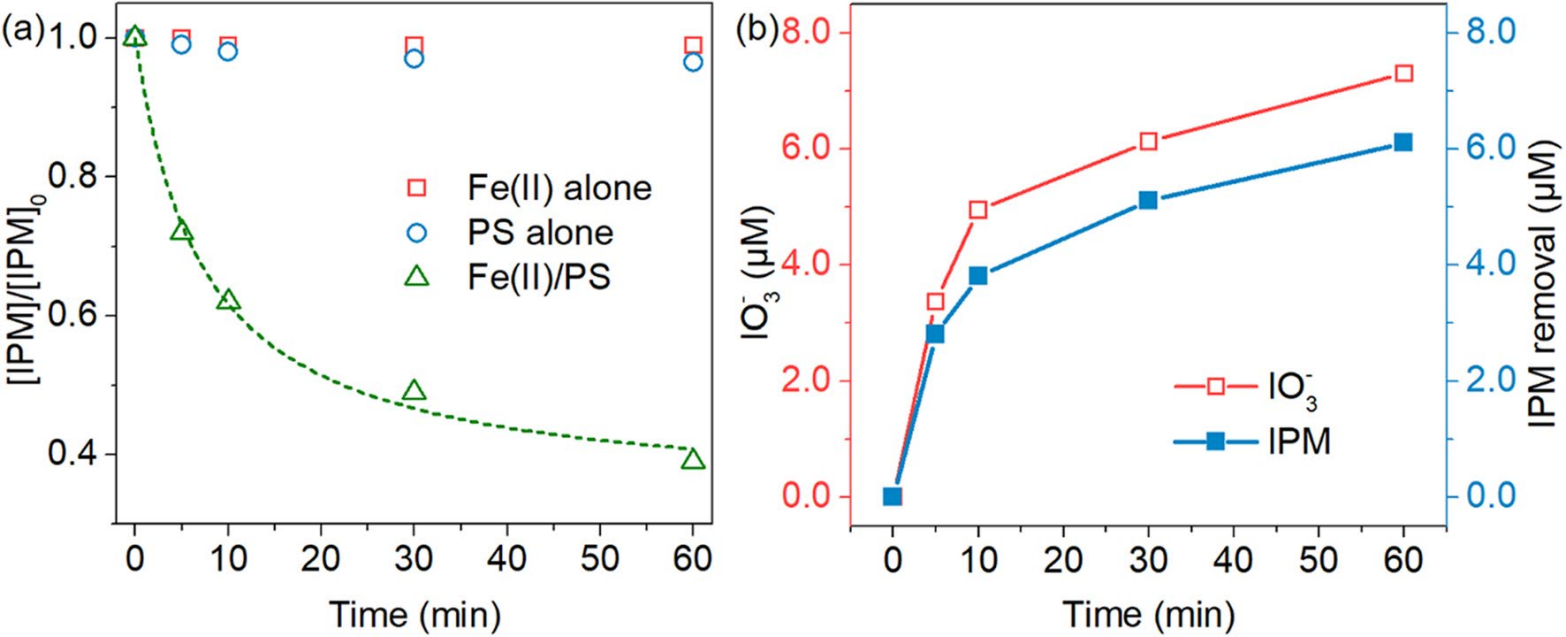
(**a**) IPM Degradation efficiency by PS alone, Fe(II) alone and Fe(II)/PS, respectively, and (**b**) the loss of iodine by Fe(II)/PS system under an experimental condition of a initial IPM concentration of 10 μM, a initial pH value of 3.0, at 25 °C, Fe(II) concentration of 0.1 mM and a PS concentration of 1 mM.

The degradation efficiency of IPM by Fe(II) , PS and Fe(II)/PS process, was carried out, and it was observed that IPM could be degrade with an obviously higher rate by Fe(II)/PS system, in contrast to Fe(II) or PS (Fig. 1a). As one kind of recalcitrant contaminant, IPM cannot be effectively degraded by sole PS or sole Fe(II), because both of them had a low oxidation ability without activation, and no more than 5% IPM was found to be removed. On the other hand, a rapid degradation of IPM in the combined system of Fe(II) and PS (Fe(II)/PS) was clearly observed, during which over 60% IPM could be degraded. These results revealed the effectiveness of Fe(II)/PS system for the removal of IPM in water. The IPM degradation profile by Fe(II)/PS system included apparently two stages, i.e. a fast IPM concentration decreasing stage followed by a slow rate, which could be well described by a model accounted for the two distinct kinetic regimes (Eq. 1)^29,30^ with R^2^ of 0.994, as follow:

1 $$\frac{{\text{C}}}{{{\text{C}}_{{0}} }}{ = 1 - }\frac{{\text{t}}}{{{{\rho + \sigma t}}}}$$

where C and C_0_ is the substrate concentration at time t and 0, respectively. ρ and σ are characteristic kinetic constant, while 1/ρ corresponds to the initial reaction rate (i.e. at t = 0), and 1/σ corresponds to the maximum conversion rate that can be achieved at the end of the reaction. The first sharp IPM degradation was probably due to Fe(II), activating a large amount of radicals to oxidize IPM with a high efficiency (Eq. 2). Afterwards, the slow IPM degradation rate might be related with the low radical concentrations after the Fe(II) activation^31^. As shown in Eqs. 3–5, the co-existence of Fe(II) and O_2_ can lead to the generation of ·OH, which could oxidize organics effectively^32^. Meanwhile, the presence of O_2_ in this system should not be ignored.

2 $${\text{Fe}}^{{2 + }} {\text{ + S}}_{{2}} {\text{O}}_{{8}}^{{2 - }} \to {\text{Fe}}^{{3 + }} {\text{ + SO}}_{{{4}^{ \cdot } }}^{ - } {\text{ + SO}}_{{4}}^{{2 - }}$$

3 $${\text{Fe}}^{{2 + }} {\text{ + O}}_{{2}} \to {\text{Fe}}^{{3 + }} {\text{ + O}}_{{{2}^{ \cdot } }}^{ - } {\text{(pH < 7)}}$$

4 $${\text{Fe}}^{{2 + }} {\text{ + {\rm O}}}_{{{2}^{ \cdot } }}^{ - } {\text{ + 2H}}^{ + } \to {\text{Fe}}^{{3 + }} {\text{ + H}}_{{2}} {\text{O}}_{{2}}$$

5 $${\text{Fe}}^{{2 + }} + {\text{H}}_{{2}} {\text{O}}_{{2}} \to {\text{Fe}}^{{3 + }} + \cdot {\text{OH + OH}}^{-}$$

Within the IMP degradation in Fe(II)/PS system, the concentration of the produced products i.e. iodate (IO_3_^−^) and iodide (I^−^) were simultaneously determined (Fig. 1b), which was consistent with the IPM removal profiles. IO_3_^−^ concentration in the bulk displayed a significant increasing trend during the reaction, while only a few amount of I^−^ was detected. Because of the quick oxidation of I^−^ into IO_3_^−^ (Fig. 1b), the IO_3_^−^ concentration in the bulk increased gradually from 0 to 7.3 μM, along with the continuous decreasing of IPM concentration, which indicates the occurrence of deiodination.

### Optimization of reaction condition parameters in Fe(II)/PS system

#### Effects of initial PS and Fe(II)/PS mole ratio

**Figure 2:**
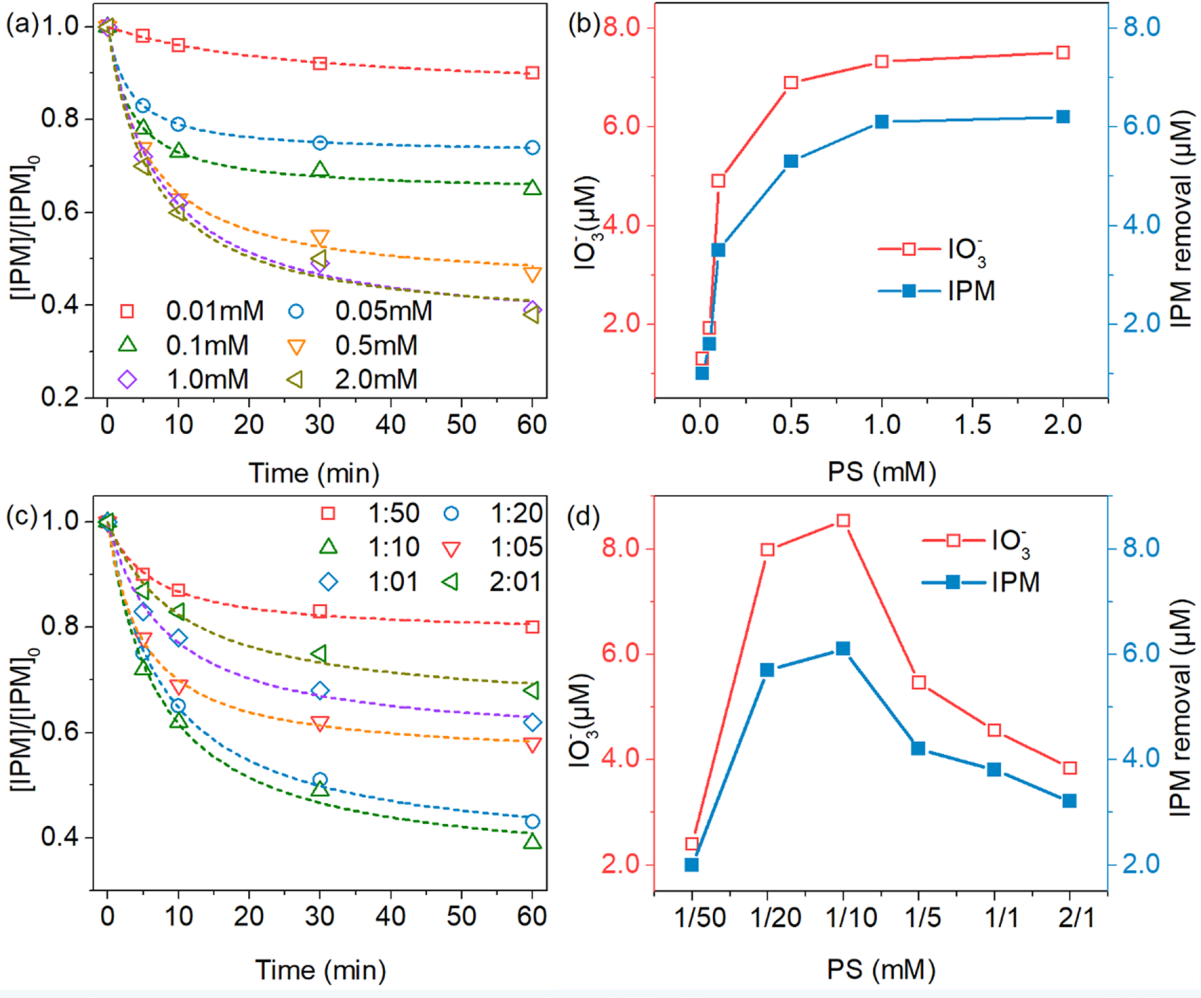
(**a**) Effect of PS concentration on IPM degradation efficiency, (**b**) the iodine amount that was removed, under an experimental condition of initial IPM concentration of 10 μM, a initial pH value of 3.0, at 25 °C and a Fe(II)/PS of 1:10, (**c**) Effect of Fe(II)/PS mole ratio on IPM degradation efficiency and (**d**) the iodine amount that was removed, under an experiment condition of a initial IPM concentration of 10 μM, a initial pH value of 3.0, at 25 °C and a PS concentration of 1 mM.

The effect of initial PS concentration (0.01, 0.05, 0.1, 0.5, 1.0 mM and 2.0 mM) on IPM degradation efficiency was carried out under a fixed Fe(II)/PS ratio, with initial pH of 3.0 at 25 °C. As the concentration of PS was increased from 0.01 mM to 1 mM, the IPM initial reaction rate was also increased from 0.0055 min^−1^ to 0.0903 min^−1^ (Fig. 2a), which was corresponded to the increased concentration of IO_3_^−^ from 1.3 μM to 7.32 μM (Fig. 2b). However, with further increased concentration of PS over 2 mM resulted in a slight improvent of degradation rate constant, as well as a similar trend was observed in the profile of the change of IO_3_^−^ concentration. The reason for this phenomenon might be due to the excess of PS which can acts as a scavenger of oxidative radicals in Fe(II)/PS system, as described in Eq. 6^33,34^.

6 $${\text{S}}_{{2}} {\text{O}}_{{8}}^{{2 - }} {\text{ + SO}}_{{{4}^{ \cdot } }}^{ - } \to {\text{SO}}_{{4}}^{{2 - }} {\text{ + S}}_{{2}} {\text{O}}_{{{8}^{ \cdot } }}^{ - } \quad k{ = 5}{\text{.5}} \times {10}^{5} \,{\text{mol}}\,{\text{L}}^{ - 1}$$

In Fe(II)/PS system, Fe(II) plays a role of an activator for the activation of PS to generate sulfate radicals, which is an important factor that controlled the overall oxidizing ability ^35^. Under a constant PS dosage of 1 mM, as showed in Fig. 2c, the rate constant for degrading IPM was improved significantly, when the initial Fe(II)/PS mole ratio increased from 1/50 to 2/1. The generated IO_3_^−^ concentration was positively correlated with the Fe(II)/PS mole ratios, which reached to the maximum of 8.54 μM when the Fe(II)/PS mole ratio increased to 1/10 (Fig. 2d). However, this IO_3_^−^ concentration began to decline once the Fe(II)/PS mole ratio increased up to 2/1, which revealed the presence of extra excessive Fe(II) preferentially reacted with SO_4_^−^· via Eq. 7. Once the SO_4_^−^· reached to a high concentration, it would be self-quenched (Eq. 8)^36^, to cause a decrease in the removal efficiency of IPM (Fig. 2d).

7 $${\text{Fe}}^{{2 + }} + {\text{SO}}_{{{4}^{\cdot}}}^{-} \to + {\text{Fe}}^{{3 + }} + {\text{SO}}_{{4}}^{{2 - }} \quad {\text{k}} = 4{{.6 \times 10}}^{{9}} \,{\text{mol}}\,{\text{L}}^{{ - 1}}$$

8 $${\text{S{O}}}_{{{4}^{ \cdot } }}^{ - } + {\text{S{O}}}_{{{4}^{ \cdot } }}^{ - } \to {\text{S}}_{{2}} {\text{O}}_{{8}}^{{2 - }} \quad {\text{k}} = 8{{.9 \times 10}}^{{8}} \,{\text{mol}}\,{\text{L}}^{{ - 1}}$$

#### Effects of initial pH and reaction temperature

**Figure 3:**
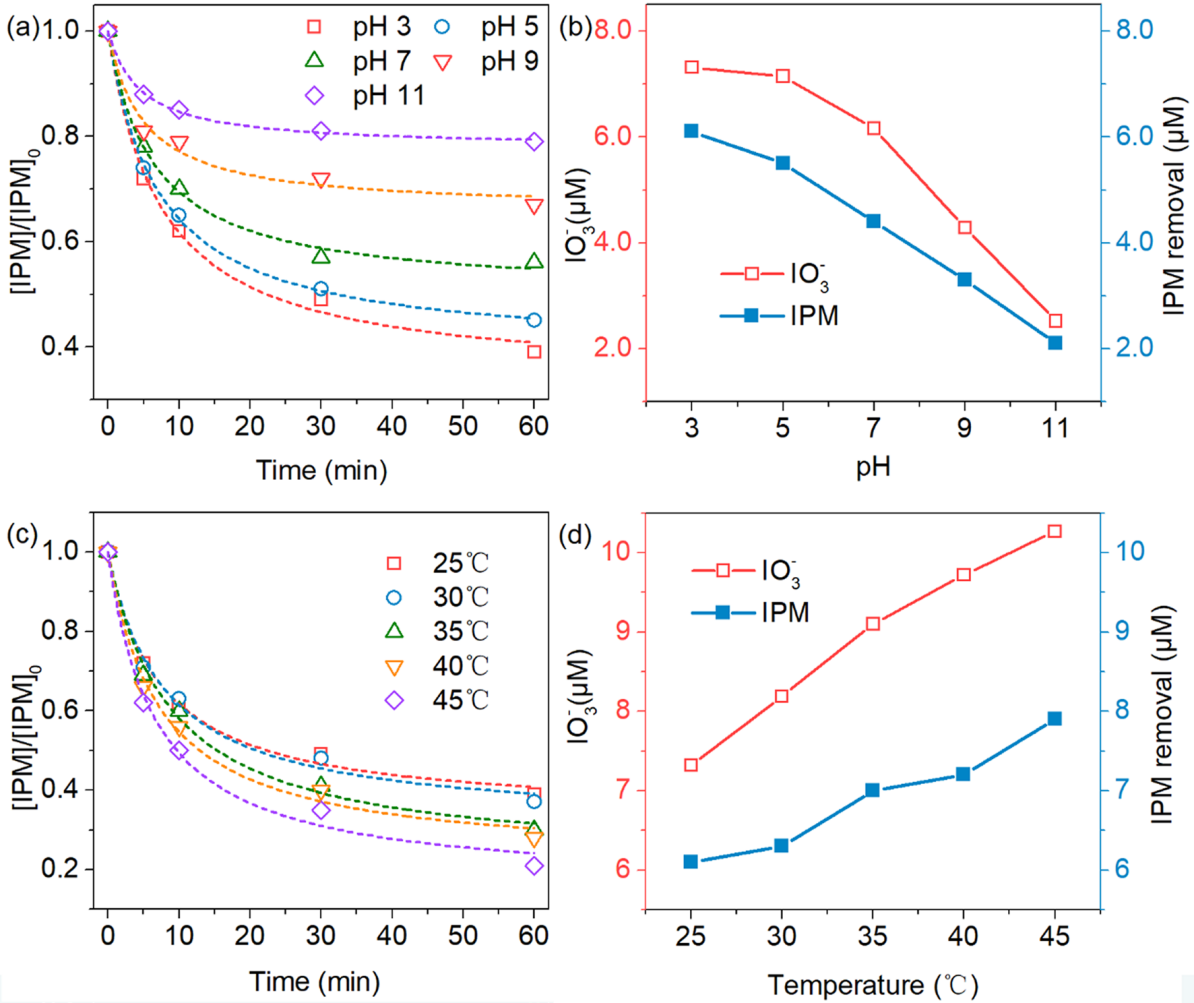
(**a**) Effect of initial solution pH on IPM degradation efficiency, (**b**) the iodine amount that was removed under an experimental condition of an initial IPM concentration of 10 μM, a PS concentration of 1 mM, at 25 °C and a Fe(II)/PS of 1:10, (**c**) Effect of reaction temperature on IPM degradation efficiency, and (**d**) the iodine amount that was removed, under an experiment condition of an initial IPM of 10 μM, a PS of 1 mM, a Fe(II)/PS of 1:10, and an initial pH of 3.0.

In all Fe based AOPs, pH is an important factor affecting the effectiveness of activation of oxidants by Fe(II) and/or Fe(III)^37^. Herein, the IPM degradation rate by activated PS was investigated at pH 3.0, 5.0, 7.0, 9.0 and 11.0, respectively, by comparing its resulted IPM degradation efficiency and IO_3_^−^ generation (Fig. 3). It was observed that the IPM initial reaction rate reached the maximum of 0.0903 min^−1^ with the initial pH of 3.0. With the increased pH value from 3.0 to 11.0, the rate constant decreased gradually from 0.0903 min^−1^ to 0.0493 min^−1^, ,which was corresponds to the reduction of IO_3_^−^ from 7.32 μM (pH = 3.0) to 3.89 μM (pH = 11.0) (Fig. 3a,b). It has been reported that PS is easily decomposed to produce SO_4_^2−^ in an alkaline solution. As shown in Eq. 9, ·OH would be generated whose oxidation–reduction potential is lower than SO_4_^−^^38^. However, sulfate and hydroxyl radicals were slightly influenced by the variation of the initial pH^38,39^. Hence, the main reason was the formation of an Fe^2+^ complex at pH > 4.0 that would be expected to hinder the reaction of Fe^2+^ with PS^39^ Hence, the oxidizing ability of the system was decreased, corresponding to a low IPM removal efficiency.

9 $${\text{SO}}_{{{4}^{ \cdot } }}^{ - } + {\text{OH}}^{ - } \to {{\cdot {\text{OH}} + {\text{SO}}}}_{{4}}^{{2 - }}$$

Besides, the variation of pH during the reaction was shown in Fig. S1, which displayed a slightly decreasing trend during IPM degradation reaction when the initial pH was 3.0. The pH level dropped significantly at the very beginning stage of the reaction, and then remained at a stable level after 5 min reaction, when the initial pH was kept above 3.0. This decreasing pH trend with the IPM degradation might be related closely to the continuous production of H^+^ in the reaction (Eq. 10)^40^.

10 $${\text{SO}}_{{{4}^{ \cdot } }}^{ - } + {\text{H}}_{2} {\text{O}} \to \cdot {\text{OH + H}}^{ + } {\text{ + SO}}_{{4}}^{{2 - }}$$

The impact of reaction temperature, i.e. 25, 30, 40 and 45 °C, on IPM degradation rate and subsequent formation of IO_3_^−^ was also evaluated, under a PS dose of 1 mM, an n(Fe(II))/n(PS) ratio of 1:10 and an initial pH of 3.0. As shown in Fig. 3c, the IPM initial reaction rate was increased from 0.0903 min^−1^ to 0.1266 min^−1^, when the temperature increased from 25 to 45 °C, during which the generated IO_3_^−^ (Fig. 3d) increased from 7.32 to 10.27 μM. An elevated temperature promotes the degradation and deiodination of IPM, which was mainly caused by the generation of more sulfate radicals. At higher temperature condition, PS would be thermally activated to produce more SO_4_^−^**·.** Moreover, the reaction of Fe(II) to activate PS is an endothermic reaction, and high reaction temperature is beneficial to this reaction, and thus to enhance the degradation and deiodination rates of IPM.

Moreover, the degradation of IPM in Fe(II)/PS system may be affected by inorganic ions and natural organic matter (NOM). Therefore, further studies on the environmental impacts would be paid more attention in future. For instance, Zhu et. al found that iohexol degradation would be promoted by the low concentration of chloride ion while inhibited by high concentration, and iohexol degradation was slightly inhibited by NOM^26^. In another study, Bu et. al found that ATZ degradation performed well at low initial ATZ and NOM concentrations^27^.

### Identification of ROS

**Figure 4:**
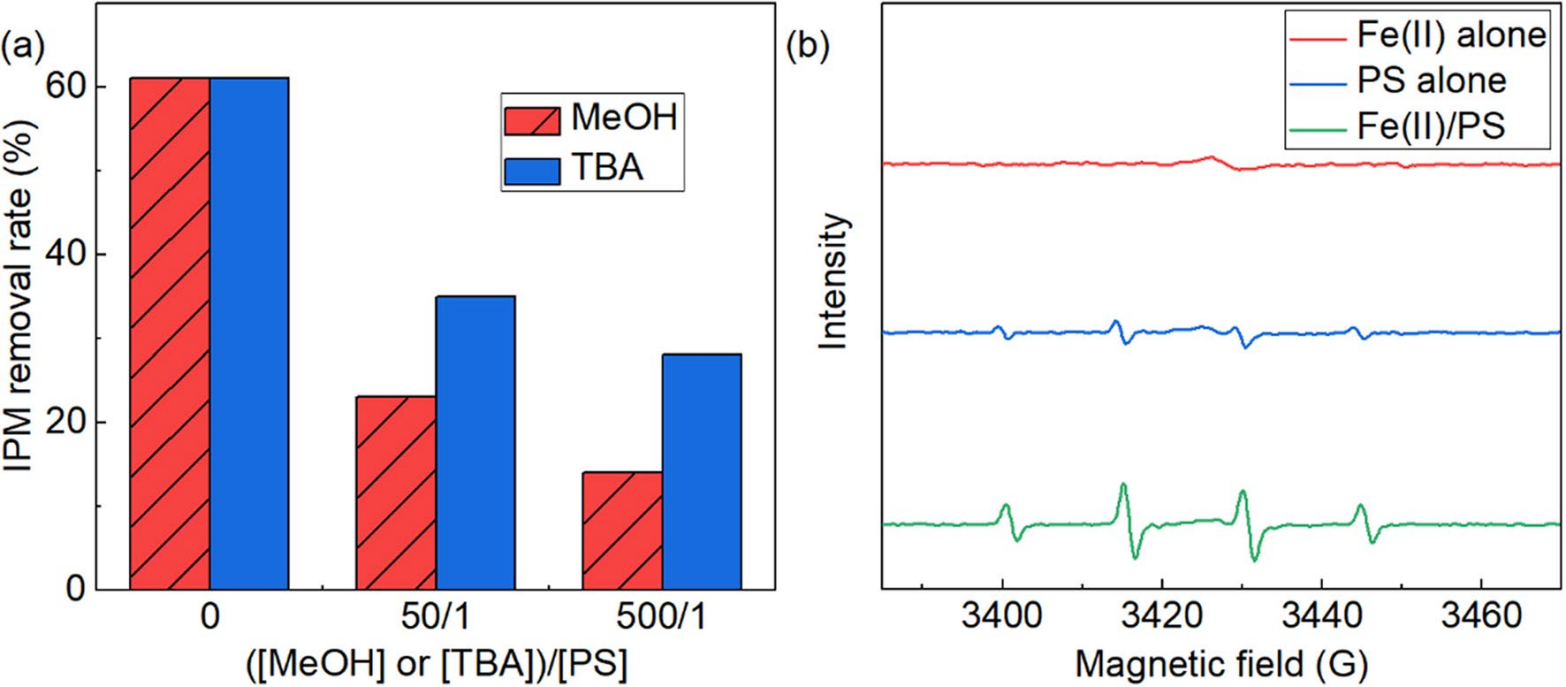
(**a**) IPM degradation efficiency with different inhibitors, and (**b**) EPR spectra of DMPO-OH· and DMPO-SO_4_·

Radical quenching experiments for the IPM degradation over the 3 samples under a pH of 3.0 were conducted to explore the catalytic mechanism during Fe(II)/PS system. Generally, it was thought that the active species SO_4_^−^**·** and **·**OH were the possible main active oxidant species in Fe(II)/PS oxidation process^27,39^. During the reaction, methanol (MeOH) was employed as the scavengers of SO_4_^−^**·** and OH**·**, while t-butanol (TBA) was applied as the inhibitor of **·**OH^41,42^. Figure 4a demonstrated the quenching experiments of active species under varied dosages of inhibitors (([MeOH] or [TBA])/[PS] was kept at 0, 50, and 500, respectively). Without any radical scavenger dosage, the IPM degradation efficiency was around 69%. In comparison, this removal efficiency decreased to 46% and 55%, when the MeOH doasges was 50 times and 500 times higher than PS concentration, respectively. Corresponding to the addition of TBA, the degradation efficiency of IPM was declined to 34% and 41%, respectively, suggesting that the **·**OH was the dominant radical in the degradation of IPM.

The EPR spin-trap technique was also carried out to unveil the reactive species in Fe(II)/PS process for IPM degradation, where 5, 5-dimethyl-1-pyrroline-N-oxide (DMPO) was used as a spin-trapping agent to capture SO_4_^−^**·** and **·**OH. As depicted in Fig. 4b, there was rather insignificant EPR signals with Fe(II) alone or PS alone, indicating rare absence of SO_4_^−^**·** and **·**OH generation. Upon the PS and Fe(II) dosage to form Fe(II)/PS oxidation process, four characteristic peaks, including a_N_ = a_H_ = 14.9 G for DMPO-OH**·** adducts, and the characteristic peaks (a_N_ = 13.9 G, a_H_ = 10 G, a_H_ = 1.48 G, a_H_ = 0.78 G) for DMPO-SO_4_^−^**·** adduct were found in the EPR spectrum, whose intensity ratio was around 1:2:2:1^43^. In addition, the DMPO- SO_4_^−^**·** adduct showed weak signals than the DMPO-OH**·** adduct, which were in accordance with the radical trapping experiments.

### IPM degradation products and reaction paths

Based on DFT quantum chemistry calculation, the degradation of IPM products and pathways in Fe(II)/PS system were analyzed. The chemical calculation is mainly based on the DFT model under Gaussian 09 where the calculation method and basis set are set for computer precision and flexibility, and comprehensive consideration of time-consuming calculations.

**Figure 5:**
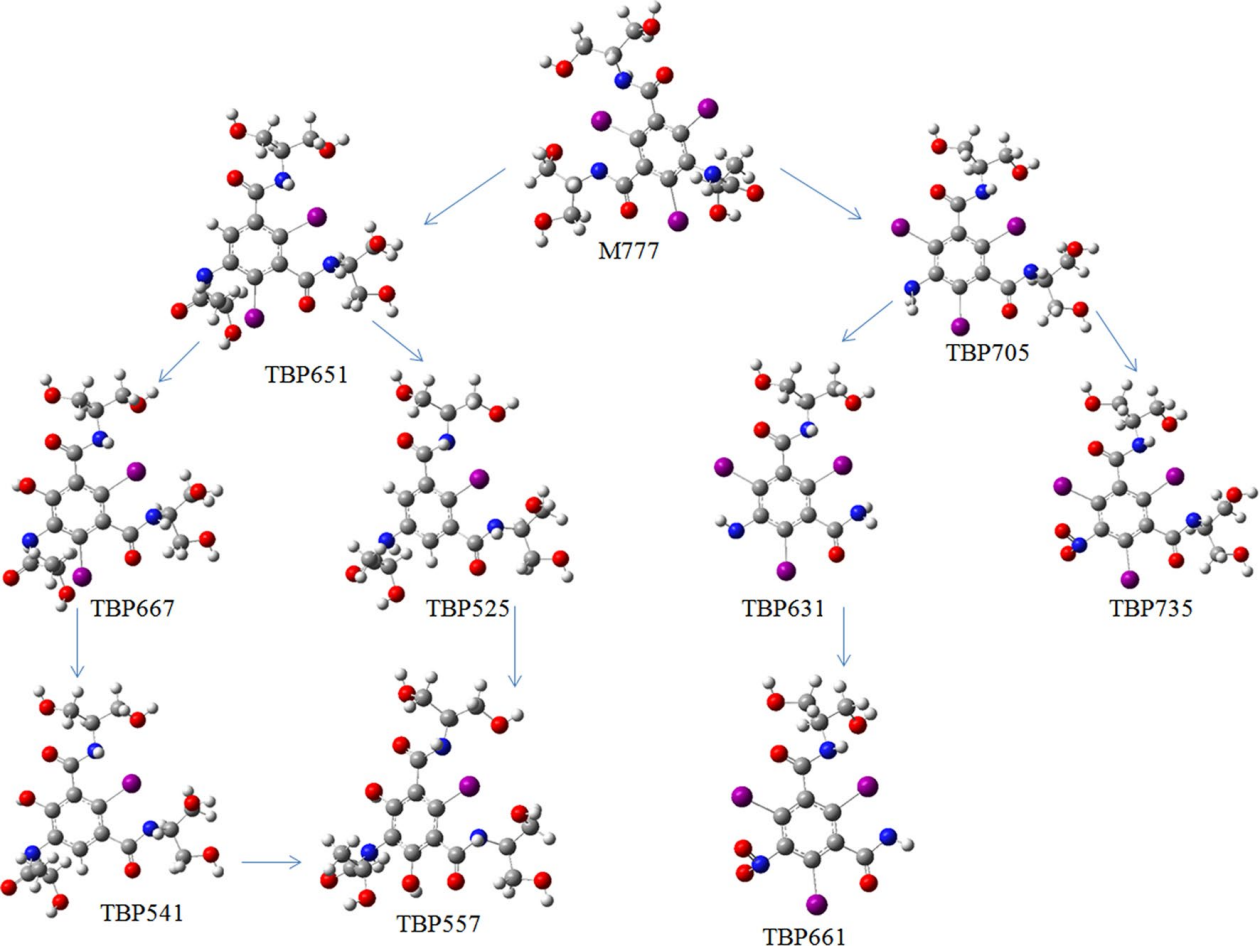
Possible degradation pathways of IPM by Fe(II)/PS system.

The main types of IPM and **·**OH radical reactions are presented in Fig. 5. Accordingly, iodine on the IPM phenyl ring would firstly be removed to produce TP651 (Eq. 11). Secondly, at this position, a hydrogen atom would be replaced by a hydroxyl group to induce hydroxylation and to produce TP667 (Eq. 12). Afterwards, the iodine at another position on the benzene ring of TP667 was removed to produce TP541 (Eq. 13), and the hydroxylation reaction occurred at the position where iodine was removed by TP541, where one hydrogen atom could be replaced by a hydroxyl group to form TP557 (Eq. 14). On the other hand, TP651 may also directly be removed iodine to produce TP525 (Eq. 15), whose benzene ring would then be hydroxylated to remove two iodines, and meanwhile the hydrogen atom could be replaced by a hydroxyl group to produce TP557 (Eq. 16). Therefore, it is reasonable to think that the IPM branch underwent a deacetylation reaction path with the action of **·**OH.

In addition, there is another possible IPM degradation path, during which the acetyl group would be deacetylated to produce TP705 (Eq. 17). Then, the branch of TP705 would be cleaved under the action of **·**OH to produce TP631 (Eq. 18), which afterwards could be oxidized by **·**OH to produce TP661 (Eq. 19). Apart from the abovementioned paths, TP705 could also directly undergo amine oxidation path under the oxidation of **·**OH, to produce TP735 (Eq. 20).11121314151617181920

## Conclusion

IPM, a typical non-ionic ICM compound, was effectively degraded by using sulfate activated PS oxidation (Fe(II)/PS) system. The effect of PS concentration, Fe(II)/PS mole ratio, initial pH, and operation temperature, on the IPM removal rate, as well as on the loss of iodine, were comprehensively investigated. The optimum degradation condition parameters were as follows: the 10 mmol L^−1^ PS concentration, 1:10 n(Fe(II))/n(PS), 3.0 pH and 45 °C temperature. The PS concentration increased from 10 to 1 mM which could enhance the IPM degradation and deiodination rates. Compared to alkaline conditions, acidic and neutral conditions were favorable for IPM degradation. The combination of SO_4_^−^**·** and **·**OH contributed to the effectiveness of IPM removal. Through DFT quantum chemical calculations, the possible reaction path of IPM degradation was mainly included hydroxylation, deiodination, deacetylation, and amine oxidation, and finally all of the iodine removed was transformed into IO_3_^−^.

Herein, this IPM initiate reaction rate of 0.1266 min^−1^ is quite higher in contrast to those listed in Table 1, under the relatively mild reaction condition. Fe(II) /PS system for IPM degradation in this study showed an excellent performance, compared to anaerobic process, PS/Fe(III) or UV system. Meanwhile, Fe(II)/PS system had a high economic feasibility, as it could degrade IPM without any additional complexion agents or expensive catalysts dosage, such as gallic acid, Fe(VI) or zero-valent aluminum. Moreover, Fe(II)/PS system possessed the advantage of sustainable, which did not need any hazardous materials and could not result in any secondary pollution. Therefore, it is reasonable to realize that, the PS/Fe(II) system reported in this study provided a sustainable approach for the practical treatment of refractory pollutants, and further studies should be carried out to evaluate its environmental impacts and stability.

## Data availability

All data or models generated or used during the study are available from the corresponding author by request.

## Supplementary information

Supplementary Figures.
