# Supplementary material for: Fe(II)-activated persulfate oxidation to degrade iopamidol in water: parameters optimization and degradation paths
Source: Sci Rep. 2020 Dec 9;10:21548. doi: 10.1038/s41598-020-78468-y (PMC7726144; doi:10.1038/s41598-020-78468-y)
Supplement: Supplementary file 1 — Supplementary Figures. [file 41598_2020_78468_MOESM1_ESM.docx]

# Supporting Information

for

**Fe(II)-activated persulfate oxidation to degrade iopamidol in water: parameters optimization and degradation paths**

Zijun Dong^1^, Guanhan Chen^2^, Mu Li^2^, Feiyun Sun^2🖂^, Chengchun Jiang^1^, & Bandna Bharti^2^

*^1^ School of Civil and Environmental Engineering, Shenzhen Polytechnic, Shenzhen 518055, China.*

*^2^ School of Civil and Environmental Engineering, Harbin Institute of Technology Shenzhen, Shenzhen 518055, China.*

*Zijun Dong and Guanhan Chen contributed equally to this work.*

^🖂^ *E-mail address*: sun_fy@hit.edu.cn





**Fig.S1** Time course of pH levels during IPM degradation reaction under an experimental conditions of IPM of 10 μM, a PS of 1 mM, a Fe(II)/PS of 1:10 and at a temperature of 25 ^o^C
